# Neutrophil-Platelet Interactions as Novel Treatment Targets in Cardiovascular Disease

**DOI:** 10.3389/fcvm.2021.824112

**Published:** 2022-01-31

**Authors:** Rainer Kaiser, Raphael Escaig, Johanna Erber, Leo Nicolai

**Affiliations:** ^1^Department of Medicine I, University Hospital, Ludwig-Maximilians University Munich, Munich, Germany; ^2^Deutsches Zentrum für Herz-Kreislauf-Forschung (DZHK, German Centre for Cardiovascular Research), Partner Site Munich Heart Alliance, Berlin, Germany; ^3^Department of Internal Medicine II, School of Medicine, University Hospital Rechts der Isar, Technical University of Munich (TUM), Munich, Germany

**Keywords:** neutrophil, platelet, cardiovascular disease, thrombosis, NETosis

## Abstract

Neutrophils and platelets are among the most abundant cell types in peripheral blood and characterized by high plasticity and a readily available reservoir of surface proteins and secretable granule contents. Receptor-mediated activation and granule release predispose both cell types for rapid responses to various stimuli. While neutrophils provide the first line of defense to microbial infections and platelets are known for their aggregatory functions in hemostasis and thrombosis, research of the past decade has highlighted that both cell types jointly shape local and systemic immune responses and clot formation alike. Concomitant activation of neutrophils and platelets has been observed in a variety of cardiovascular diseases, including arterial and venous thrombosis, atherosclerosis as well as myocardial infarction and ischemia-reperfusion injury. In this review, we describe the mechanisms by which neutrophils and platelets interact physically, how release of granule contents and soluble molecules by either cell type affects the other and how this mutual activation supports the efficacy of immune responses. We go on to describe how activated platelets contribute to host defense by triggering neutrophil extracellular trap (NET) formation in a process termed immunothrombosis, which in turn promotes local platelet activation and coagulation. Further, we review current evidence of hazardous overactivation of either cell type and their respective role in cardiovascular disease, with a focus on thrombosis, myocardial infarction and ischemia-reperfusion injury, and describe how neutrophils and platelets shape thromboinflammation in COVID-19. Finally, we provide an overview of therapeutic approaches targeting neutrophil-platelet interactions as novel treatment strategy in cardiovascular disease.

## Introduction

Neutrophils and platelets are among the most abundant cells in peripheral blood. Traditionally, a clear division of labor was proposed between these cell types: one providing the most essential of host defenses against invading pathogens (1), the other forming plugs to prevent bleeding (2). Evolutionarily, inflammatory reactions to invading pathogens and hemostatic responses share common features: Both infection and impairment of vascular integrity observed in sterile injury lead to exposure of damage- or pathogen-associated molecular patterns (PAMPs and DAMPs), respectively, the sensing of which results in immune cell activation, cytokine and granule release and concurrent effects on vascular homeostasis (3, 4). Consequently, the traditional view of platelets and neutrophils acting separately has started to blur, with both clinical and experimental studies pointing toward the involvement of both cell types in coagulation and immunity alike (5, 6). Interestingly, neutrophils and platelets show significant overlap of surface receptors such as Toll-like Receptors (TLR) (1, 7–9) capable of sensing DAMPs and PAMPs, and both possess an intracellular weaponry of granules, ready to be released upon detection of pathogens or loss of endothelial integrity, respectively.

In this review, we describe the armamentarium of both platelets and neutrophils comprising multiple surface receptors and soluble agonists that shape the intricate interplay between both cell types. We go on to describe the effects of reciprocal platelet-neutrophil activation observed in inflammation and its impact on immune responses and clot formation alike. Specifically, we focus on platelet-neutrophil interactions and their hazardous overactivation in cardiovascular disease, with a focus on atherosclerosis, thrombosis, and ischemia-reperfusion injury (IRI). We then provide an overview on how detrimental platelet and neutrophil overactivation and exhaustion drive cardiovascular complications and a prothrombotic phenotype observed in severe Coronavirus disease 2019 (COVID-19). Finally, we summarize currently investigated therapeutic approaches that target platelet-neutrophil interactions.

## Mechanisms of Platelet-Neutrophil Interplay

### Direct Interactions

**Figure 1:**
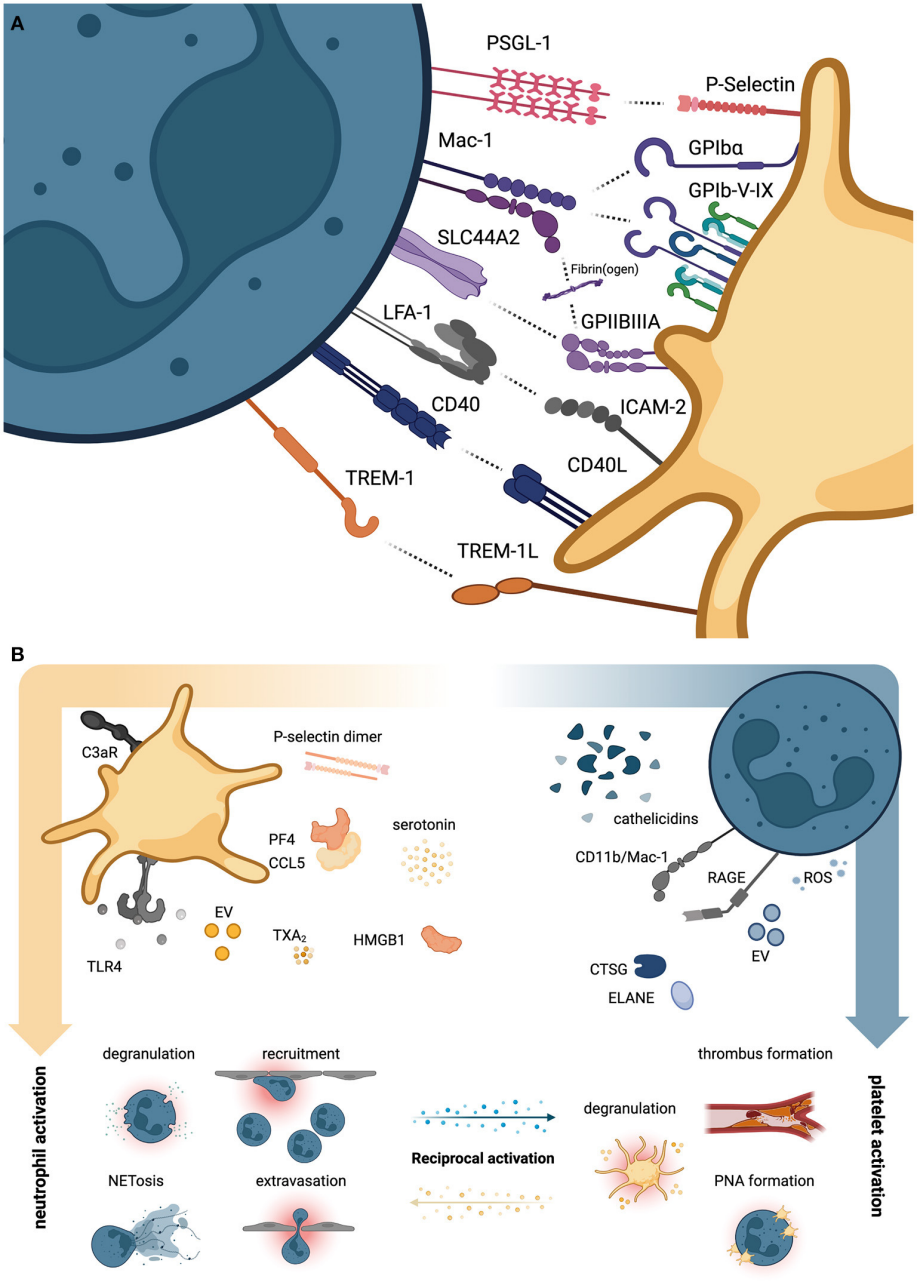
Direct and indirect platelet-neutrophil interactions. **(A)** Overview of neutrophil (left, petrol) and platelet receptor pairs (right, yellow) that directly engage and promote reciprocal activation of either cell type. **(B)** Overview of soluble agonists secreted by platelets promoting neutrophil activation (left panel) and vice versa (right panel). CTSG, cathepsin G; ELANE, neutrophil elastase; EV, extracellular vesicles; HMGB1, High mobility group box 1; ROS, reactive oxygen species; TXA_2_, thromboxane A_2_. Created with BioRender.com.

P-selectin on platelets and P-selectin glycoprotein ligand-1 (PSGL-1) on neutrophils are among the most important molecules mediating platelet-neutrophil interaction. Together with glycoprotein (GP) Ibα and the neutrophil integrin αMβ2, known as Mac-1 (CD11b/CD18), this receptor pair drives platelet-neutrophil interactions under (thrombo)inflammatory conditions (Figure 1A) (10–12). Indeed, adherent neutrophils were shown to actively scan flowing blood for activated platelets using PSGL-1-positive uropods to subsequently transmigrate across the inflamed endothelium (11). Mac-1 also mediates neutrophil-platelet interactions through a third receptor expressed on platelets, the junctional adhesion molecule 3 (JAM-3) (13).

Interestingly, P-selectin-induced neutrophil activation supports neutrophilic secretion of cathepsin G (CTSG) and neutrophil elastase (ELANE), both of which cleave the N-terminus of surface PSGL-1, thereby negatively regulating further activation (12). Direct physical interactions between platelets and neutrophils are also mediated by CD40 ligand (CD40L/CD154) and CD40 (14), the adhesion-molecule ICAM-2 and integrin αLβ2, known as Lymphocyte function-associated antigen 1 (LFA-1) (5), and TREM-1 ligand (TREM-1L) and TREM-1 (15) on platelets and neutrophils, respectively (Figure 1A). Binding *via* these receptors can induce reciprocal activation of downstream signaling cascades: For e.g., TREM-1L/TREM-1 binding promotes neutrophil reactive oxygen species (ROS) production, degranulation and release of IL-8 (15, 16), without affecting platelet aggregation (15), while CD40-CD40L interaction boosts platelet-neutrophil interaction, platelet activation and enhances neutrophil recruitment through upregulation of Mac-1 (17, 18).

Notably, well-known platelet adhesion receptors GPIb-V-IX complex and integrin αIIbβ3, known as GPIIBIIIA, which mediate binding of extracellular matrix and plasma proteins such as von Willebrand factor (vWF) and fibrin(ogen), were shown to also confer platelet-neutrophil interactions through Mac-1. The immunoreceptor tyrosine-based activation motif (ITAM) receptor GPVI, the main platelet receptor for collagen, aids in local and systemic immune responses by promoting platelet-neutrophil aggregate formation and neutrophil recruitment in a model of gram-negative, pneumonia-driven sepsis (19). A recent study found that septic patients requiring intensive care treatment displayed acquired dysfunctions in GPVI-mediated platelet activation (20).

### Interactions Through Secreted Molecules and Cytokines

While not directly mediating platelet-neutrophil interactions, numerous receptors are implicated in activation of platelets, which subsequently promote neutrophil binding and propagation of activation. These receptors include TLRs like TLR4, which promote both neutrophil activation and neutrophil extracellular trap (NET) formation in endotoxemia and gram-negative sepsis (8), complement receptors such as C3aR, which enhance thromboinflammation and neutrophil-mediated damage in myocardial infarction (21), and eicosanoid receptors (Figure 1B). Regarding the latter, a seminal study recently described the shuttling of neutrophil-derived arachidonic acid into platelets in a P-selectin-dependent manner, which was processed to thromboxane A2 (TXA_2_) by activated platelets (22). Subsequent TXA_2_ secretion induced endothelial cell activation, ICAM-1 expression and promoted neutrophil recruitment to sites of inflammation. Consequently, targeting of platelet activation through blockade of soluble agonist receptors such as the adenosine-diphosphate (ADP) receptor P2Y_12_ reduced platelet-neutrophil interaction and attenuated thromboinflammation in endotoxemia (23, 24).

Platelet secretion of several soluble mediators affects neutrophil functions under steady-state conditions and inflammation alike: Platelet-derived heterodimers consisting of platelet factor 4 (PF4) and CCL5/RANTES (regulated on activation, normal T cell expressed and secreted) stored in alpha granules (2, 25) promote neutrophil extravasation in acute lung injury (26) and are known to modulate neutrophil function in sterile inflammation (27). Similarly, soluble P-selectin dimers shed by activated platelets promote leukocyte adhesion and NET formation under inflammatory conditions in mice (28). Further, High mobility group box 1 (HMGB1) secreted by activated platelets enhances neutrophil recruitment by activating Receptor for advanced glycation endproducts (RAGE) on neutrophils (29). Similarly, serotonin stored in platelet dense granules promotes neutrophil CD11b expression, oxidative bursts and degranulation in mice and men (30). Vice-versa, neutrophil-derived agonists such as cathelicidins (LL37 in humans, CRAMP in mice) can activate platelets and thereby propagate thrombus formation, as shown in mouse models of arterial thrombosis and histopathological analyses of human thrombi (31). Further, enzymes such as ELANE and CTSG as well as ROS released by neutrophils promote platelet activation (32) through cleavage of protease-activated receptors (PAR), as recently shown for CTSG and PAR4 (33). This reciprocal influence of both cell types is also observed at the level of extracellular vesicles (EVs): While platelet-derived EVs promote NET formation in septic shock and dengue virus infection (34, 35) and are able to shuttle regulatory RNA to neutrophils (36), neutrophil-derived EVs transfer metabolites to platelets and thereby promote reciprocal activation (22).

In summary, platelets and neutrophils interact and co-operate through a vast variety of receptors, soluble agonists and even EV-mediated shuttling of effector molecules. Since some of the secreted agonists mentioned above are especially released after physical interaction of both cell types, direct binding and subsequent agonist release may promote reciprocal (hyper)activation at both the local and the systemic level (5, 37).

## Reciprocal Platelet and Neutrophil Activation Drives Immunothrombosis

Reciprocal binding of platelets and neutrophils leads to phenotypical changes in both cell types. Classical neutrophil responses upon platelet binding include conformational changes of adhesion and activation receptors such as CD11b and CD177, but also shedding of surface molecules like L-selectin (CD62L) (1). In neutrophils, MAPK- and Syk-mediated signaling upon platelet interaction mediates production and release of the neutrophil chemoattractant CXCL8/interleukin-8 (IL-8) and beta-integrin activation, enhancing neutrophil recruitment and adhesion to ICAM-1-positive endothelial cells (38).

Functionally, platelet binding can boost phagocytic capacity and bacterial clearance exerted by neutrophils, as well as production and ROS secretion under inflammatory conditions (11, 39). Immuno-responsive platelets also collect and bundle pathogens, aiding in local control of infection (40). Consequently, thrombocytopenia is associated with reduced neutrophil activation and impaired bacterial clearance (41). One of the most striking features of platelet-neutrophil interactions is the ability of platelets to trigger neutrophil extracellular trap (NET) formation, a specific death program by which neutrophils release nuclear DNA covered with histones and neutrophil effector enzymes into blood and the interstitium (41). This process, which is triggered by P-selectin/PSGL-1 interactions (42) and likely represents an ancient defense mechanism to prevent spreading of a variety of pathogens including bacteria, viruses, and fungi (8, 35, 43–47), enables neutrophils to “ensnare” circulating microbes and facilitates clearance by phagocytes (8, 41, 48). Consequently, pharmacological or genetic ablation of NET formation or cleavage of NETs by exogenous DNases aggravates systemic bacterial load and is associated with worsening outcome in models of bacterial infection (8, 46, 48). The complex molecular mechanisms underlying NET formation and resolution are discussed elsewhere (37, 49, 50).

In addition to facilitating pathogen capture, NET formation also promotes coagulation through a plethora of mechanisms like histone-mediated platelet activation, cleavage of tissue factor pathway inhibitor (TFPI) and thrombomodulin by effector enzymes like ELANE and CTSG, and direct binding of vWF and factor XII (46, 49, 51–53). *In vivo* live imaging studies of liver sinusoids have shown increased thrombin turnover locally associated with NETs, suggesting a direct stimulation of plasmatic coagulation through NETosis (54). However, only isolated DNA and histones, but not intact NETs, were shown to drive activation of the contact pathway and thrombin generation *in vitro* (55). Mechanistically, neutralization of negative charges of supercoiled DNA through histone/nucleosome binding is thought to underly the lack of procoagulant potential observed in isolated NETs (55).

The pathophysiological process that underlies inflammation-driven NETosis, recruitment of proinflammatory monocytes, platelet activation and increased coagulation with deposition of fibrin(ogen) is termed immunothrombosis. Immunothrombosis is considered a protective host defense mechanism designated to prevent microbial spreading (49). While enhancing pathogen capture during infection, dysregulated and excessive activation of immunothrombosis—termed thromboinflammation—can promote local ischemia and subsequent collateral organ damage. In addition, thromboinflammation has been attributed to remote organ injury, aggravation of systemic inflammation and disseminated intravascular coagulation (DIC) (37, 49, 56–59). In addition, excessive NET formation may be detrimental in some cases of bacterial infection: In models of infective endocarditis, NETs were shown to induce trapping and formation of platelet-bacteria aggregates on injured heart valves, thereby potentiating bacterial growth and vegetation expansion (60, 61).

## Platelet-Neutrophil Interactions in Cardiovascular Disease

### Chronic (Cardio)Vascular Inflammation

Cardiovascular events including myocardial infarction, ischemia-driven heart failure and stroke are all sequelae of atherosclerosis and remain the most frequent cause of death world-wide (62).

**Figure 2:**
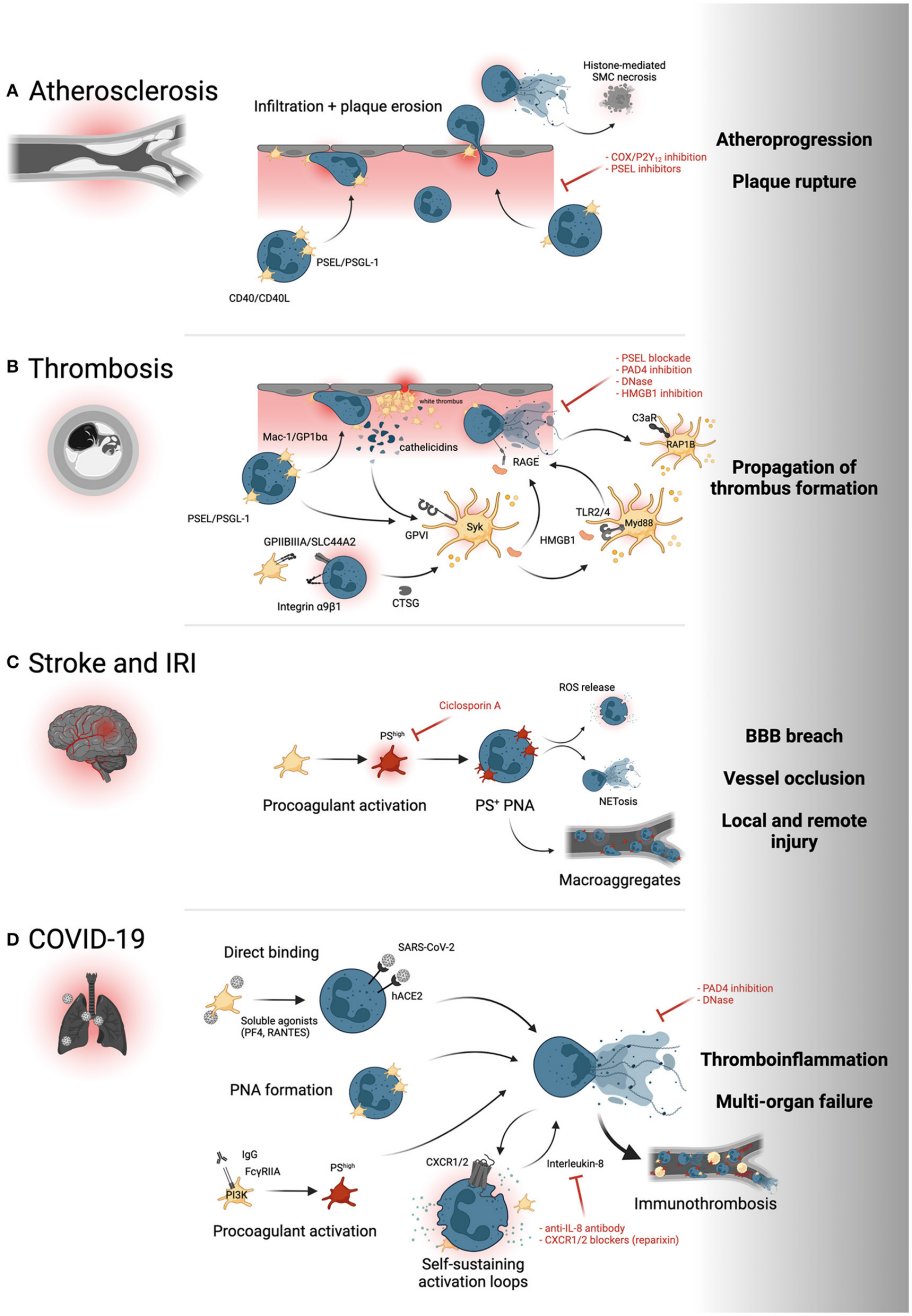
Platelet-neutrophil interactions in cardiovascular disease. **(A)** PSEL/PSGL-1- and CD40L/CD40-dependent recruitment of neutrophils to the atherosclerotic plaque promotes neutrophil infiltration and subsequent destabilization of the plaque, for instance through histone-mediated cell death of smooth muscle cells (SMC). **(B)** Both arterial and venous thrombosis are promoted by PNA formation and reciprocal activation of platelets and neutrophils through GPVI/cathelicidin, GPIIBIIIA/SLC44A2, HMGB1/RAGE, HMGB1/TLR and complement/C3aR interactions. Syk, Syk family kinase; RAP1B, Ras-related protein Rap-1b; Myd88, Myeloid differentiation primary response 88. **(C)** Inflammatory responses in stroke and ischemia-reperfusion injury induce procoagulant platelet activation (red) and formation of phosphatidylserine (PS)^+^ PNAs that promote neutrophil activation and infiltration and propagate thrombus formation through platelet-neutrophil macroaggregates. **(D)** Direct binding of SARS-CoV-2 to either platelets or neutrophils, subsequent PNA formation and hyperactivation, IgG-mediated procoagulant platelet activation and IL-8 mediated neutrophil self-stimulation all promote NET formation and systemic thromboinflammation associated with severe COVID-19. PI3K, Phosphoinositide 3-kinase; CXCR1/2, C-X-C motif chemokine receptor 1/2. Yellow intravascular cells depict proinflammatory monocytes. Created with BioRender.com.

Since the early 2000s, evidence has emerged highlighting the role of platelet adhesion and subsequent leukocyte—and specifically neutrophil—recruitment in early atherogenesis (63) and the progression of atherosclerotic lesions (64, 65). This evidence is supported by more recent data that implicate a general role for platelets in atheroprogression through recruitment of other leukocyte subsets—namely monocytes and eosinophils (66, 67). Both neutrophilia and thrombocytosis as well as increased platelet-neutrophil aggregates (PNA) are observed in patients with chronic cardiovascular conditions such as peripheral (PAD) (68) and coronary artery disease (CAD) (69–71). Local recruitment of circulating neutrophils to atherosclerotic plaques is enhanced by platelets binding to inflamed endothelium (Figure 2A) (14, 72). Subsequent PNA formation has been shown to depend on P-selectin/PSGL-1 interaction, and elevated soluble P-selectin is observed in patients with cardiovascular disease and positively correlates with the risk of major adverse cardiovascular events (MACE) (73–75). In addition, CD40-CD40L interactions of platelets and neutrophils and other leukocyte subsets exacerbates atherosclerosis, while CD40 deficiency was associated with reduced atheroprogression (14). Enhanced adhesion of neutrophils to sites with high atherosclerotic plaque burden as well as neutrophil recruitment into the plaque are associated with plaque instability, erosion, and rupture (76–78). Activated neutrophils recruited into the plaque can exacerbate chronic vascular inflammation, specifically atherogenesis and atheroprogression, by NET formation, promoting a proinflammatory macrophage phenotype and inducing histone H4-mediated lytic cell death of smooth muscle cells inside the plaque (79, 80). Targeting endovascular platelet-neutrophil interactions before subsequent neutrophil adhesion and plaque recruitment ensue therefore provides therapeutic promise. The effects of platelet-neutrophil interactions in other entities of chronic cardiovascular inflammation, such as abdominal aortic aneurysms, are reviewed elsewhere (81, 82).

### Thrombosis

As a consequence of atheroprogression, plaque erosion and—ultimately—plaque rupture, thrombogenic material including vWF, extracellular matrix proteins and cellular debris released by the necrotic core is exposed to circulating blood cells (76). Sensing of PAMPs and extracellular matrix proteins swiftly leads to the recruitment of platelets *via* GPIbα-vWF and GPVI–collagen interactions (2), which start to form a cellular clot called the “white thrombus” (83). These highly activated platelets recruit leukocytes in a Mac-1/GPIbα- and PSGL-1/P-selectin-dependent manner, promoting fibrin(ogen) deposition and enhancing clot stability (10, 84). Notably, the recruitment cascade appears to be model-dependent, and leukocyte recruitment to laser-induced arterial injury was shown to depend on LFA-1/ICAM-1 neutrophil-endothelium interactions, but not solely on platelets (85). Within this prothrombotic microenvironment, a self-sustaining loop of reciprocal platelet and neutrophil activation is initiated (Figure 2B): Activated neutrophils secrete cathelicidins (LL-37/CRAMP), which promote further platelet activation and aggregation through the collagen receptor GPVI and its downstream signaling cascades, including tyrosine kinase Syk (31). Next, platelets secrete HMGB1, which self-stimulates platelets *via* the TLR4-Myd88 axis and promotes NET formation through RAGE on neutrophils, thereby sustaining platelet activation and clot stability (29, 39). Platelet-neutrophil crosstalk in arterial thrombosis is enhanced by two additional mechanisms: A recent study revealed that neutrophilic α9β1 mediates platelet activation and arterial thrombus formation. Mechanistically, α9β1-promoted platelet activation reciprocally enhances NET formation and secretion of CTSG, which in turn sustains arterial neutrophil recruitment (86, 87). Of note, blockade of platelet-induced NETosis through pharmacological or genetic ablation of Protein arginine deiminase 4 (PAD4) attenuates arterial thrombus formation (78, 88). Following neutrophil activation, excreted NETs promote platelet activation and aggregation through complement C3 activation and subsequent platelet signaling through the anaphylatoxin receptor C3aR and downstream RAP1B (21, 89). Consequently, C3aR deficiency reduces platelet activation and attenuates arterial thrombosis (90). The translational relevance of these observations in mouse models of arterial thrombosis are underlined by the presence of LL-37 and TF-positive NETs in coronary artery thrombi from patients with acute myocardial infarction and a marked NET signature in thrombi from patients suffering from coronary stent thromboses (31, 91–93).

In venous thrombosis, endothelial inflammatory responses and ICAM-1/VECAM expression promote swift recruitment of platelet and both monocytes and neutrophils alike and the formation of PNAs and platelet-monocyte aggregates (PMAs) (37, 94). The underlying molecular mechanisms are reminiscent of arterial thrombosis and involve PSEL/PSGL-1 interactions, HMGB1-induced platelet and neutrophil activation through TLR2/4 and RAGE, respectively, and propagation of immunothrombosis through NET formation (29, 37). A novel interaction between activated GPIIBIIIA on platelets and the choline transporter SLC44A2 on neutrophils was recently observed under low shear rate. This interaction enhanced NET formation and thereby propagated deep vein thrombosis (95). Supporting the role of neutrophils and platelets in venous thrombosis, both genetic ablation of P-selectin, blockade of HMGB1 receptors and therapeutic targeting of NETs through exogenous DNases or genetic ablation of PAD4 resolved thrombus formation (52, 94, 96).

### Ischemic Stroke and Ischemia-Reperfusion Injury

Ischemic stroke, triggered by either cardiac embolism or carotid plaque rupture, is characterized by systemic increases in circulating neutrophils and formation of platelet-neutrophil aggregates, with the extent of neutrophilia correlating with infarction size and patient prognosis (97, 98). In a pivotal study, Denorme et al. (99) described a hazardous interplay between neutrophils and a hyperactivated subtype of platelets, so-called procoagulant platelets, in mice. This platelet population, which is induced upon combined stimulation of either strong soluble agonists or high mechanical shear stress, is hallmarked by high levels of phosphatidylserine (PS) exposure, microvesiculation and striking morphological changes, namely platelet ballooning (100–103). In a model of ischemia-reperfusion injury (IRI) of the brain, procoagulant platelets were induced systemically, subsequently promoted recruitment of neutrophils across the blood-brain-barrier (BBB), leading to intracerebral ROS release, NETosis and exacerbated brain damage (Figure 2C). Targeting procoagulant platelet activation through genetic ablation or by pharmacological blockade of PS attenuated cerebral damage and enhanced neurological outcomes (99, 104, 105).

Platelet-neutrophil interplay also affects IRI in other organs, such as heart, liver and kidney, as described elsewhere (106–109). Of note, reperfusion injury in the lung is attributed to platelet-neutrophil interplay: While platelet-dependent NET formation was observed in experimental models and clinical samples of lung transplant dysfunction (110), Yuan et al. found that gut ischemia induced the formation of PS^+^ platelet-neutrophil aggregates in the mesenterial vasculature, which subsequently led to uncontrolled NETosis and the formation of platelet-neutrophil macroaggregates occluding the lung vasculature (111). Targeting either platelet-neutrophil interactions or platelet-induced NETosis alleviated intravascular thrombosis and enhanced outcomes, suggesting PNA and NET formation as potent clinical targets.

## COVID-19-Related Immunothrombosis

The COVID-19 pandemic has impacted global human life, and an unprecedented research effort has been initiated to understand and counter this viral infection caused by SARS-CoV-2. Severe cases are defined by hyperinflammation reminiscent of cytokine release syndromes (112). Interestingly, patients with cardiovascular comorbidities are at high risk of developing severe COVID-19, which is associated with an increased incidence of venous and arterial thrombotic complications like pulmonary embolism and myocardial infarction (113–115). Indeed, a systemic procoagulant state correlating with disease severity was identified in COVID-19 patients (57). As outlined above, thrombosis in inflammatory disease has been linked to intravascular interplay of platelet and innate immune cells in the framework of immunothrombosis (46, 116). Along these lines, thrombocytosis and neutrophilia were described in severe cases of SARS-CoV-2 infection, and both cell types showed unique activation patterns compared to healthy controls and other viral infections (37, 49, 56–59).

Mechanistically, this immunothrombotic state seems to at least in part be mediated by neutrophil-platelet interplay: *In vitro*, addition of platelets derived from severe COVID-19 patients to healthy neutrophils induced activation and NETosis (57). This seems to be triggered by direct interactions, but also by the release of soluble mediators like RANTES and PF4 by platelets, known to be involved in NET formation (Figure 2D) (117). Platelet transcriptomics revealed upregulated protein ubiquitination, antigen presentation, and mitochondrial dysfunction in COVID-19 patients (118). This coincided with increased MAPK signaling and higher numbers of circulating platelet-leukocyte aggregates (PLA), including PNAs (118). In summary, platelets are primed to interact and trigger neutrophil activation in SARS-CoV-2 infection, which in turn leads to neutrophil activation, granule release, and NETosis, triggering immunothrombotic dysregulation. Preceding platelet activation seems to be induced by three key events: An important mechanism is vascular inflammation and endotheliopathy induced by this betacoronavirus, which leads to endothelial disruption and platelet activation in the vasculature (59, 116, 119). Second, direct association and uptake of SARS-CoV-2 viral particles by platelets is observed, which triggers hyperactivation (118, 120). Third, a research group identified increased procoagulant potential indicated by PS exposure and intracellular calcium elevation, induced by autoantibodies and driven by the phosphoinositid-3-kinase (PI3K)/protein kinase B (AKT) signaling pathway (121, 122). Interestingly, procoagulant function was induced by Fcγ receptor IIA binding of plasmatic IgG antibody fractions of COVID-19 patients (121). Procoagulant platelets are also known to preferentially interact with neutrophils, contributing to thromboinflammation in experimental stroke, possibly contributing to the increase in thrombotic events in patients with severe courses (99). In conclusion, platelet-neutrophil interplay is crucial in shaping the prothrombotic phenotype observed in COVID-19 and might offer a path to therapeutic intervention.

## Therapeutic Targeting of Platelet-Neutrophil Interactions

As outlined above, targeting platelet-neutrophil interplay might be a valuable tool to fight cardiovascular disease, in particular acute thromboinflammation. One promising approach are the P-selectin antibodies inclacumab and crizanlizumab that block interaction of platelet and endothelial P-Selectin with neutrophilic PSGL-1. *In vitro* experiments confirmed that inclacumab inhibits PLA formation (123). A landmark study, SELECT-ACS, showed that inclacumab at a dosing of 20 mg/kg significantly reduced myocardial damage assessed by CK-MB levels after percutaneous coronary intervention in patients with non-ST-segment elevation myocardial infarction (124, 125). In contrast, the SELECT-CABG Trial showed no effect of inclacumab on bypass graft failure after coronary bypass (126). Crizanlizumab, also blocking PSGL-1/P-selectin interaction, was shown to be safe and effective at preventing vasoocclusive crises mediated by platelet-neutrophil interactions in sickle cell disease (SUSTAIN study) (127). Importantly, receiving anti-P-selectin antibodies was not associated with increased infection or bleeding rates, while other adverse events like diarrhea and chest pain exceeded those observed in placebo-treated patients (124–127). Further studies are needed to assess the potential of these antibodies in acute and chronic coronary syndromes. Another study targeting PLAs in pneumonia with Ticagrelor (XANTHIPPE) (128) revealed that ticagrelor reduced the fraction of platelet-bound leukocytes and IL-6 levels, and also decreased oxygen demand compared to placebo-treated patients without affecting the incidence of adverse events. Ticagrelor has been shown to be superior to clopidogrel in preventing death from vascular causes, myocardial infarction, or stroke, but the mechanisms are not fully understood and might involve reductions in circulating PLAs (129). In experimental settings, additional therapeutic approaches that focus on interference with other platelet receptors like GPIbα have been shown to decrease neutrophil recruitment into inflamed tissue (130).

Targeting leukocyte integrins has proven a valuable therapeutic approach for refractory chronic inflammatory diseases (131, 132), and therapeutic interference with neutrophil Mac-1 attenuates both inflammation and thrombosis in experimental settings (10, 133, 134). As outlined above, NETosis is a common effector mechanism of neutrophils triggered by platelets and holds an important role in mediating intravascular thrombosis and clot formation. Therefore, pharmacological inhibition and degradation of NETs represents a promising approach in a range of acute and chronic inflammatory conditions. The anticoagulant heparin, used widely in acute thrombotic events, disrupts NETs and neutralizes histones, which contributes to its clinical effect (135, 136). In cystic fibrosis patients, treatment with inhaled NET degrading rhDNase1, known as dornase alfa, was associated with improved oxygenation and decreased DNA:MPO complexes in BALF (137). Ongoing studies are addressing its effects in COVID-19 (NCT04409925) and ischemic stroke (NCT04785066).

PF4 and CCL5/RANTES are important platelet chemokines shaping innate intravascular immune responses. The antiretroviral drug Maraviroc targets the CCL5 receptor CCR5, and was developed as an entry inhibitor against HIV, since CCR5 is a co-receptor for viral entry (138). In addition to its antiviral properties, Maraviroc was found to reduce PLAs and markers of atherosclerosis like carotid intima-media thickness in HIV patients (139). This was also confirmed in an experimental mouse model of atherosclerosis (140). A naturally occurring loss-of-function mutation in CCR5 was also found to be protective after stroke and traumatic brain injury, however, these effects seem to also directly influence neurons beyond affecting platelet-leukocyte interplay (141). Studies addressing the efficacy of Maraviroc in stroke have been initiated (NCT04789616).

## Conclusions

Recent evidence has highlighted novel interaction pathways between neutrophils and platelets that crucially effect acute thromboinflammation and chronic vascular disease alike. Further translational studies as well as clinical trials will help to pin down the most effective therapeutic strategies depending on disease manifestation and patient characteristics.

## Author Contributions

RK, RE, and LN reviewed the literature and wrote the initial draft. RK and JE prepared the figures. All authors reviewed and approved the final version of the manuscript.

## Funding

This study was supported by Deutsche Forschungsgemeinschaft [Clinican Scientist Program PRIME 413635475 (RK)], Deutsche Herzstiftung e.V., Frankfurt a. M. (RK), and the German Center for Cardiovascular Research [DZHK, Clinician Scientist Programme (LN), Shared Expertise Grant (RK and LN)].

## Conflict of Interest

The authors declare that the research was conducted in the absence of any commercial or financial relationships that could be construed as a potential conflict of interest.

## Publisher's Note

All claims expressed in this article are solely those of the authors and do not necessarily represent those of their affiliated organizations, or those of the publisher, the editors and the reviewers. Any product that may be evaluated in this article, or claim that may be made by its manufacturer, is not guaranteed or endorsed by the publisher.

## References

[1] Burn GL Foti A Marsman G Patel DF Zychlinsky A. The neutrophil. Immunity. (2021) 54:1377–91. 10.1016/j.immuni.2021.06.006 34260886

[2] van der Meijden PEJ Heemskerk JWM. Platelet biology and functions: new concepts and clinical perspectives. Nat Rev Cardiol. (2019) 16:166–79. 10.1038/s41569-018-0110-0 30429532

[3] Silvis MJM Kaffka Genaamd Dengler SE Odille CA Mishra M van der Kaaij NP Doevendans PA. Damage-Associated molecular patterns in myocardial infarction and heart transplantation: the road to translational success. Front Immunol. (2020) 11:599511. 10.3389/fimmu.2020.599511 33363540 PMC7752942

[4] Hotchkiss RS Moldawer LL Opal SM Reinhart K Turnbull IR Vincent JL. Sepsis and septic shock. Nat Rev Dis Primers. (2016) 2:16045. 10.1038/nrdp.2016.45 28117397 PMC5538252

[5] Nicolai L Gaertner F Massberg S. Platelets in host defense: experimental and clinical insights. Trends Immunol. (2019) 40:922–38. 10.1016/j.it.2019.08.004 31601520

[6] Gaertner F Massberg S. Patrolling the vascular borders: platelets in immunity to infection and cancer. Nat Rev Immunol. (2019) 19:747–60. 10.1038/s41577-019-0202-z 31409920

[7] D'Atri LP Schattner M. Platelet toll-like receptors in thromboinflammation. Front Biosci. (2017) 22:1867–83. 10.2741/4576 28410150

[8] Clark SR Ma AC Tavener SA McDonald B Goodarzi Z Kelly MM. Platelet TLR4 activates neutrophil extracellular traps to ensnare bacteria in septic blood. Nat Med. (2007) 13:463–9. 10.1038/nm1565 17384648

[9] Prince LR Whyte MK Sabroe I Parker LC. The role of TLRs in neutrophil activation. Curr Opin Pharmacol. (2011) 11:397–403. 10.1016/j.coph.2011.06.007 21741310

[10] Wang Y Gao H Shi C Erhardt PW Pavlovsky A D AS. Leukocyte integrin Mac-1 regulates thrombosis *via* interaction with platelet GPIbalpha. Nat Commun. (2017) 8:15559. 10.1038/ncomms15559 28555620 PMC5477519

[11] Sreeramkumar V Adrover JM Ballesteros I Cuartero MI Rossaint J Bilbao I. Neutrophils scan for activated platelets to initiate inflammation. Science. (2014) 346:1234–8. 10.1126/science.1256478 25477463 PMC4280847

[12] Gardiner EE De Luca M McNally T Michelson AD Andrews RK Berndt MC. Regulation of P-selectin binding to the neutrophil P-selectin counter-receptor P-selectin glycoprotein ligand-1 by neutrophil elastase and cathepsin *Blood*. (2001) 98:1440–7. 10.1182/blood.V98.5.1440 11520793

[13] Santoso S Sachs UJ Kroll H Linder M Ruf A Preissner KT. The junctional adhesion molecule 3 (JAM-3) on human platelets is a counterreceptor for the leukocyte integrin Mac-1. J Exp Med. (2002) 196:679–91. 10.1084/jem.20020267 12208882 PMC2194005

[14] Gerdes N Seijkens T Lievens D Kuijpers MJ Winkels H Projahn D. Platelet CD40 exacerbates atherosclerosis by transcellular activation of endothelial cells and leukocytes. Arterioscler Thromb Vasc Biol. (2016) 36:482–90. 10.1161/ATVBAHA.115.307074 26821950

[15] Haselmayer P Grosse-Hovest L von Landenberg P Schild H Radsak MP. TREM-1 ligand expression on platelets enhances neutrophil activation. Blood. (2007) 110:1029–35. 10.1182/blood-2007-01-069195 17452516

[16] Radsak MP Salih HR Rammensee HG Schild H. Triggering receptor expressed on myeloid cells-1 in neutrophil inflammatory responses: differential regulation of activation and survival. J Immunol. (2004) 172:4956–63. 10.4049/jimmunol.172.8.4956 15067076

[17] Rahman M Zhang S Chew M Ersson A Jeppsson B Thorlacius H. Platelet-derived CD40L (CD154) mediates neutrophil upregulation of Mac-1 and recruitment in septic lung injury. Ann Surg. (2009) 250:783–90. 10.1097/SLA.0b013e3181bd95b7 19806052

[18] Vanichakarn P Blair P Wu C Freedman JE Chakrabarti S. Neutrophil CD40 enhances platelet-mediated inflammation. Thromb Res. (2008) 122:346–58. 10.1016/j.thromres.2007.12.019 18289643

[19] Claushuis TAM de Vos AF Nieswandt B Boon L Roelofs J de Boer OJ. Platelet glycoprotein VI aids in local immunity during pneumonia-derived sepsis caused by gram-negative bacteria. Blood. (2018) 131:864–76. 10.1182/blood-2017-06-788067 29187378

[20] Weiss LJ Manukjan G Pflug A Winter N Weigel M Nagler N. Acquired platelet GPVI receptor dysfunction in critically ill patients with sepsis. Blood. (2021) 137:3105–15. 10.1182/blood.2020009774 33827131

[21] Sauter RJ Sauter M Reis ES Emschermann FN Nording H Ebenhoch S. Functional relevance of the anaphylatoxin receptor c3ar for platelet function and arterial thrombus formation marks an intersection point between innate immunity and thrombosis. Circulation. (2018) 138:1720–35. 10.1161/CIRCULATIONAHA.118.034600 29802205 PMC6202244

[22] Rossaint J Kuhne K Skupski J Van Aken H Looney MR Hidalgo A. Directed transport of neutrophil-derived extracellular vesicles enables platelet-mediated innate immune response. Nat Commun. (2016) 7:13464. 10.1038/ncomms13464 27845343 PMC5116072

[23] Totani L Dell'Elba G Martelli N Di Santo A Piccoli A Amore C. Prasugrel inhibits platelet-leukocyte interaction, reduces inflammatory markers in a model of endotoxic shock in the mouse. Thromb Haemost. (2012) 107:1130–40. 10.1160/TH11-12-0867 22436970

[24] Hagiwara S Iwasaka H Hasegawa A Oyama M Imatomi R Uchida T. Adenosine diphosphate receptor antagonist clopidogrel sulfate attenuates LPS-induced systemic inflammation in a rat model. Shock. (2011) 35:289–92. 10.1097/SHK.0b013e3181f48987 20720514

[25] Harrison P Cramer EM. Platelet alpha-granules. Blood Rev. (1993) 7:52–62. 10.1016/0268-960X(93)90024-X 8467233

[26] Grommes J Alard JE Drechsler M Wantha S Morgelin M Kuebler WM. Disruption of platelet-derived chemokine heteromers prevents neutrophil extravasation in acute lung injury. Am J Respir Crit Care Med. (2012) 185:628–36. 10.1164/rccm.201108-1533OC 22246174 PMC3326286

[27] Vajen T Koenen RR Werner I Staudt M Projahn D Curaj A. Blocking CCL5-CXCL4 heteromerization preserves heart function after myocardial infarction by attenuating leukocyte recruitment and NETosis. Sci Rep. (2018) 8:10647. 10.1038/s41598-018-29026-0 30006564 PMC6045661

[28] Panicker SR Mehta-D'souza P Zhang N Klopocki AG Shao B McEver RP. Circulating soluble P-selectin must dimerize to promote inflammation and coagulation in mice. Blood. (2017) 130:181–91. 10.1182/blood-2017-02-770479 28515093 PMC5510792

[29] Stark K Philippi V Stockhausen S Busse J Antonelli A Miller M. Disulfide HMGB1 derived from platelets coordinates venous thrombosis in mice. Blood. (2016) 128:2435–49. 10.1182/blood-2016-04-710632 27574188 PMC5147023

[30] Mauler M Herr N Schoenichen C Witsch T Marchini T Hardtner C. Platelet serotonin aggravates myocardial ischemia/reperfusion injury *via* neutrophil degranulation. Circulation. (2019) 139:918–31. 10.1161/CIRCULATIONAHA.118.033942 30586717 PMC6370531

[31] Pircher J Czermak T Ehrlich A Eberle C Gaitzsch E Margraf A. Cathelicidins prime platelets to mediate arterial thrombosis and tissue inflammation. Nat Commun. (2018) 9:1523. 10.1038/s41467-018-03925-2 29670076 PMC5906636

[32] Renesto P Chignard M. Enhancement of cathepsin G-induced platelet activation by leukocyte elastase: consequence for the neutrophil-mediated platelet activation. Blood. (1993) 82:139–44. 10.1182/blood.V82.1.139.bloodjournal821139 8324217

[33] Stoller ML Basak I Denorme F Rowley JW Alsobrooks J Parsawar K. Neutrophil cathepsin g proteolysis of protease activated receptor 4 generates a novel, functional tethered ligand. Blood Adv. (2021). 10.1182/bloodadvances.2021006133. [Epub ahead of print]. Available online at: https://ashpublications.org/bloodadvances/article/doi/10.1182/bloodadvances.2021006133/483040/Neutrophil-Cathepsin-G-Proteolysis-of-Protease PMC9006282 34883511

[34] Jiao Y Li W Wang W Tong X Xia R Fan J. Platelet-derived exosomes promote neutrophil extracellular trap formation during septic shock. Crit Care. (2020) 24:380. 10.1186/s13054-020-03082-3 32600436 PMC7322900

[35] Sung PS Huang TF Hsieh SL. Extracellular vesicles from CLEC2-activated platelets enhance dengue virus-induced lethality *via* CLEC5A/TLR2. Nat Commun. (2019) 10:2402. 10.1038/s41467-019-10360-4 31160588 PMC6546763

[36] Fendl B Eichhorn T Weiss R Tripisciano C Spittler A Fischer MB. Differential interaction of platelet-derived extracellular vesicles with circulating immune cells: roles of TAM receptors, CD11b, and phosphatidylserine. Front Immunol. (2018) 9:2797. 10.3389/fimmu.2018.02797 30619243 PMC6297748

[37] Stark K Massberg S. Interplay between inflammation and thrombosis in cardiovascular pathology. Nat Rev Cardiol. (2021) 18:666–82. 10.1038/s41569-021-00552-1 33958774 PMC8100938

[38] Urzainqui A Serrador JM Viedma F Yanez-Mo M Rodriguez A Corbi AL. ITAM-based interaction of ERM proteins with Syk mediates signaling by the leukocyte adhesion receptor PSGL-1. Immunity. (2002) 17:401–12. 10.1016/S1074-7613(02)00420-X 12387735

[39] Maugeri N Campana L Gavina M Covino C De Metrio M Panciroli C. Activated platelets present high mobility group box 1 to neutrophils, inducing autophagy and promoting the extrusion of neutrophil extracellular traps. J Thromb Haemost. (2014) 12:2074–88. 10.1111/jth.12710 25163512

[40] Nicolai L Schiefelbein K Lipsky S Leunig A Hoffknecht M Pekayvaz K. Vascular surveillance by haptotactic blood platelets in inflammation and infection. Nat Commun. (2020) 11:5778. 10.1038/s41467-020-19515-0 33188196 PMC7666582

[41] Brinkmann V Reichard U Goosmann C Fauler B Uhlemann Y Weiss DS. Neutrophil extracellular traps kill bacteria. Science. (2004) 303:1532–5. 10.1126/science.1092385 15001782

[42] Etulain J Martinod K Wong SL Cifuni SM Schattner M Wagner DD. P-selectin promotes neutrophil extracellular trap formation in mice. Blood. (2015) 126:242–6. 10.1182/blood-2015-01-624023 25979951 PMC4497964

[43] Koupenova M Corkrey HA Vitseva O Manni G Pang CJ Clancy L. The role of platelets in mediating a response to human influenza infection. Nat Commun. (2019) 10:1780. 10.1038/s41467-019-09607-x 30992428 PMC6467905

[44] Jenne CN Wong CH Zemp FJ McDonald B Rahman MM Forsyth PA. Neutrophils recruited to sites of infection protect from virus challenge by releasing neutrophil extracellular traps. Cell Host Microbe. (2013) 13:169–80. 10.1016/j.chom.2013.01.005 23414757

[45] Gaertner F Ahmad Z Rosenberger G Fan S Nicolai L Busch B. Migrating platelets are mechano-scavengers that collect and bundle bacteria. Cell. (2017) 171:1368–1382 e23. 10.1016/j.cell.2017.11.001 29195076

[46] Massberg S Grahl L von Bruehl ML Manukyan D Pfeiler S Goosmann C. Reciprocal coupling of coagulation and innate immunity *via* neutrophil serine proteases. Nat Med. (2010) 16:887–96. 10.1038/nm.2184 20676107

[47] Branzk N Lubojemska A Hardison SE Wang Q Gutierrez MG Brown GD. Neutrophils sense microbe size and selectively release neutrophil extracellular traps in response to large pathogens. Nat Immunol. (2014) 15:1017–25. 10.1038/ni.2987 25217981 PMC4236687

[48] McDonald B Urrutia R Yipp BG Jenne CN Kubes P. Intravascular neutrophil extracellular traps capture bacteria from the bloodstream during sepsis. Cell Host Microbe. (2012) 12:324–33. 10.1016/j.chom.2012.06.011 22980329

[49] Engelmann B Massberg S. Thrombosis as an intravascular effector of innate immunity. Nat Rev Immunol. (2013) 13:34–45. 10.1038/nri3345 23222502

[50] Papayannopoulos V. Neutrophil extracellular traps in immunity and disease. Nat Rev Immunol. (2018) 18:134–47. 10.1038/nri.2017.105 28990587

[51] Semeraro F Ammollo CT Morrissey JH Dale GL Friese P Esmon NL. Extracellular histones promote thrombin generation through platelet-dependent mechanisms, involvement of platelet TLR2 and TLR4. Blood. (2011) 118:1952–61. 10.1182/blood-2011-03-343061 21673343 PMC3158722

[52] Brill A Fuchs TA Chauhan AK Yang JJ De Meyer SF Kollnberger M. von Willebrand factor-mediated platelet adhesion is critical for deep vein thrombosis in mouse models. Blood. (2011) 117:1400–7. 10.1182/blood-2010-05-287623 20959603 PMC3056477

[53] Fuchs TA Brill A Duerschmied D Schatzberg D Monestier M Myers DD Jr Wrobleski SK. Extracellular DNA traps promote thrombosis. Proc Natl Acad Sci USA. (2010) 107:15880–5. 10.1073/pnas.1005743107 20798043 PMC2936604

[54] McDonald B Davis RP Kim SJ Tse M Esmon CT Kolaczkowska E. Platelets and neutrophil extracellular traps collaborate to promote intravascular coagulation during sepsis in mice. Blood. (2017) 129:1357–67. 10.1182/blood-2016-09-741298 28073784 PMC5345735

[55] Noubouossie DF Whelihan MF Yu YB Sparkenbaugh E Pawlinski R Monroe DM. *In vitro* activation of coagulation by human neutrophil DNA and histone proteins but not neutrophil extracellular traps. Blood. (2017) 129:1021–9. 10.1182/blood-2016-06-722298 27919911 PMC5324715

[56] Nicolai L Leunig A Brambs S Kaiser R Joppich M Hoffknecht ML. Vascular neutrophilic inflammation and immunothrombosis distinguish severe COVID-19 from influenza pneumonia. J Thromb Haemost. (2021) 19:574–81. 10.1111/jth.15179 33217134 PMC7753335

[57] Nicolai L Leunig A Brambs S Kaiser R Weinberger T Weigand M. Immunothrombotic dysregulation in COVID-19 pneumonia is associated with respiratory failure and coagulopathy. Circulation. (2020) 142:1176–89. 10.1161/CIRCULATIONAHA.120.048488 32755393 PMC7497892

[58] Gando S Levi M Toh CH. Disseminated intravascular coagulation. Nat Rev Dis Primers. (2016) 2:16037. 10.1038/nrdp.2016.37 27250996

[59] Kaiser R Leunig A Pekayvaz K Popp O Joppich M Polewka V. Self-sustaining IL-8 loops drive a prothrombotic neutrophil phenotype in severe COVID-19. JCI Insight. (2021) 6:e150862. 10.1172/jci.insight.150862 34403366 PMC8492337

[60] Jung CJ Yeh CY Hsu RB Lee CM Shun CT Chia JS. Endocarditis pathogen promotes vegetation formation by inducing intravascular neutrophil extracellular traps through activated platelets. Circulation. (2015) 131:571–81. 10.1161/CIRCULATIONAHA.114.011432 25527699

[61] Hsu CC Hsu RB Ohniwa RL Chen JW Yuan CT Chia JS. Neutrophil extracellular traps enhance staphylococcus aureus vegetation formation through interaction with platelets in infective endocarditis. Thromb Haemost. (2019) 119:786–796. 10.1055/s-0039-1678665 30731490

[62] GBD 2019 Diseases and Injuries Collaborators. Global burden of 369 diseases and injuries in 204 countries and territories, 1990-2019: a systematic analysis for the global burden of disease study 2019. Lancet. (2020) 396:1204–22. 10.1016/S0140-6736(20)30925-9 33069326 PMC7567026

[63] Gawaz M Langer H May AE. Platelets in inflammation and atherogenesis. J Clin Invest. (2005) 115:3378–84. 10.1172/JCI27196 16322783 PMC1297269

[64] Huo Y Schober A Forlow SB Smith DF Hyman MC Jung S. Circulating activated platelets exacerbate atherosclerosis in mice deficient in apolipoprotein E. Nat Med. (2003) 9:61–7. 10.1038/nm810 12483207

[65] Massberg S Brand K Gruner S Page S Muller E Muller I. A critical role of platelet adhesion in the initiation of atherosclerotic lesion formation. J Exp Med. (2002) 196:887–96. 10.1084/jem.20012044 12370251 PMC2194025

[66] Marx C Novotny J Salbeck D Zellner KR Nicolai L Pekayvaz K. Eosinophil-platelet interactions promote atherosclerosis and stabilize thrombosis with eosinophil extracellular traps. Blood. (2019) 134:1859–72. 10.1182/blood.2019000518 31481482 PMC6908806

[67] Barrett TJ Schlegel M Zhou F Gorenchtein M Bolstorff J Moore KJ. Platelet regulation of myeloid suppressor of cytokine signaling 3 accelerates atherosclerosis. Sci Transl Med. (2019) 11:eaax0481. 10.1126/scitranslmed.aax0481 31694925 PMC6905432

[68] Dopheide JF Rubrech J Trumpp A Geissler P Zeller GC Bock K. Leukocyte-platelet aggregates-a phenotypic characterization of different stages of peripheral arterial disease. Platelets. (2016) 27:658–67. 10.3109/09537104.2016.1153619 27352829

[69] Nijm J Wikby A Tompa A Olsson AG Jonasson L. Circulating levels of proinflammatory cytokines and neutrophil-platelet aggregates in patients with coronary artery disease. Am J Cardiol. (2005) 95:452–6. 10.1016/j.amjcard.2004.10.009 15695127

[70] Furman MI Benoit SE Barnard MR Valeri CR Borbone ML Becker RC. Increased platelet reactivity and circulating monocyte-platelet aggregates in patients with stable coronary artery disease. J Am Coll Cardiol. (1998) 31:352–8. 10.1016/S0735-1097(97)00510-X 9462579

[71] Shah D Denaxas S Nicholas O Hingorani AD Hemingway H. Neutrophil counts and initial presentation of 12 cardiovascular diseases: a CALIBER cohort study. J Am Coll Cardiol. (2017) 69:1160–9. 10.1016/j.jacc.2016.12.022 28254179 PMC5332591

[72] Lievens D von Hundelshausen P. Platelets in atherosclerosis. Thromb Haemost. (2011) 106:827–38. 10.1160/TH11-08-0592 22012554

[73] Libby P Buring JE Badimon L Hansson GK Deanfield J Bittencourt MS. Atherosclerosis. Nat Rev Dis Primers. (2019) 5:56. 10.1038/s41572-019-0106-z 31420554

[74] Blann D Nadar SK Lip GY. The adhesion molecule P-selectin and cardiovascular disease. Eur Heart J. (2003) 24:2166–79. 10.1016/j.ehj.2003.08.021 14659768

[75] Ridker PM Buring JE Rifai N. Soluble P-selectin and the risk of future cardiovascular events. Circulation. (2001) 103:491–5. 10.1161/01.CIR.103.4.491 11157711

[76] Fahed C Jang IK. Plaque erosion and acute coronary syndromes: phenotype, molecular characteristics and future directions. Nat Rev Cardiol. (2021) 18:724–34. 10.1038/s41569-021-00542-3 33953381

[77] Silvestre-Roig C Braster Q Ortega-Gomez A Soehnlein O. Neutrophils as regulators of cardiovascular inflammation. Nat Rev Cardiol. (2020) 17:327–40. 10.1038/s41569-019-0326-7 31996800

[78] Franck G Mawson TL Folco EJ Molinaro R Ruvkun V Engelbertsen D. Roles of PAD4 and NETosis in experimental atherosclerosis and arterial injury: implications for superficial erosion. Circ Res. (2018) 123:33–42. 10.1161/CIRCRESAHA.117.312494 29572206 PMC6014872

[79] Silvestre-Roig C Braster Q Wichapong K Lee EY Teulon JM Berrebeh N. Externalized histone H4 orchestrates chronic inflammation by inducing lytic cell death. Nature. (2019) 569:236–240. 10.1038/s41586-019-1167-6 31043745 PMC6716525

[80] Warnatsch A Ioannou M Wang Q Papayannopoulos V. Inflammation. Neutrophil extracellular traps license macrophages for cytokine production in atherosclerosis. Science. (2015) 349:316–20. 10.1126/science.aaa8064 26185250 PMC4854322

[81] Soehnlein O Libby P. Targeting inflammation in atherosclerosis - from experimental insights to the clinic. Nat Rev Drug Discov. (2021) 20:589–610. 10.1038/s41573-021-00198-1 33976384 PMC8112476

[82] Schrottmaier WC Mussbacher M Salzmann M Assinger A. Platelet-leukocyte interplay during vascular disease. Atherosclerosis. (2020) 307:109–20. 10.1016/j.atherosclerosis.2020.04.018 32439204

[83] Jackson SP. Arterial thrombosis–insidious, unpredictable and deadly. Nat Med. (2011) 17:1423–36. 10.1038/nm.2515 22064432

[84] Palabrica T Lobb R Furie BC Aronovitz M Benjamin C Hsu YM. Leukocyte accumulation promoting fibrin deposition is mediated *in vivo* by P-selectin on adherent platelets. Nature. (1992) 359:848–51. 10.1038/359848a0 1279433

[85] Darbousset R Thomas GM Mezouar S Frere C Bonier R Mackman N. Tissue factor-positive neutrophils bind to injured endothelial wall and initiate thrombus formation. Blood. (2012) 120:2133–43. 10.1182/blood-2012-06-437772 22837532

[86] Dhanesha N Nayak MK Doddapattar P Jain M Flora GD Kon S. Targeting myeloid-cell specific integrin alpha9beta1 inhibits arterial thrombosis in mice. Blood. (2020) 135:857–61. 10.1182/blood.2019002846 31951649 PMC7068033

[87] Ortega-Gomez A Salvermoser M Rossaint J Pick R Brauner J Lemnitzer P. Cathepsin G controls arterial but not venular myeloid cell recruitment. Circulation. (2016) 134:1176–88. 10.1161/CIRCULATIONAHA.116.024790 27660294 PMC5288007

[88] Liu Y Carmona-Rivera C Moore E Seto NL Knight JS Pryor M. Myeloid-Specific deletion of peptidylarginine deiminase 4 mitigates atherosclerosis. Front Immunol. (2018) 9:1680. 10.3389/fimmu.2018.01680 30140264 PMC6094966

[89] Schreiber A Rousselle A Becker JU von Massenhausen A Linkermann A Kettritz R. Necroptosis controls NET generation and mediates complement activation, endothelial damage, autoimmune vasculitis. Proc Natl Acad Sci USA. (2017) 114:E9618–E9625. 10.1073/pnas.1708247114 29078325 PMC5692554

[90] Gushiken FC Han H Li J Rumbaut RE Afshar-Kharghan V. Abnormal platelet function in C3-deficient mice. J Thromb Haemost. (2009) 7:865–70. 10.1111/j.1538-7836.2009.03334.x 19291167 PMC2867673

[91] Riegger J Byrne RA Joner M Chandraratne S Gershlick AH Ten Berg JM. Histopathological evaluation of thrombus in patients presenting with stent thrombosis. A multicenter European study: a report of the prevention of late stent thrombosis by an interdisciplinary global European effort consortium. Eur Heart J. (2016) 37:1538–49. 10.1093/eurheartj/ehv419 26761950 PMC4872283

[92] Stakos DA Kambas K Konstantinidis T Mitroulis I Apostolidou E Arelaki S. Expression of functional tissue factor by neutrophil extracellular traps in culprit artery of acute myocardial infarction. Eur Heart J. (2015) 36:1405–14. 10.1093/eurheartj/ehv007 25660055 PMC4458286

[93] Mangold A Alias S Scherz T Hofbauer M Jakowitsch J Panzenbock A. Coronary neutrophil extracellular trap burden and deoxyribonuclease activity in ST-elevation acute coronary syndrome are predictors of ST-segment resolution and infarct size. Circ Res. (2015) 116:1182–92. 10.1161/CIRCRESAHA.116.304944 25547404

[94] von Bruhl ML Stark K Steinhart A Chandraratne S Konrad I Lorenz M. Monocytes, neutrophils, and platelets cooperate to initiate and propagate venous thrombosis in mice *in vivo*. J Exp Med. (2012) 209:819–35. 10.1084/jem.20112322 22451716 PMC3328366

[95] Constantinescu-Bercu A Grassi L Frontini M Salles C II Woollard K Crawley JT. Activated alphaIIbbeta3 on platelets mediates flow-dependent NETosis *via* SLC44A2. Elife. (2020) 9:e53353. 10.7554/eLife.53353 32314961 PMC7253179

[96] Martinod K Demers M Fuchs TA Wong SL Brill A Gallant M. Neutrophil histone modification by peptidylarginine deiminase 4 is critical for deep vein thrombosis in mice. Proc Natl Acad Sci US A. (2013) 110:8674–9. 10.1073/pnas.1301059110 23650392 PMC3666755

[97] Kim J Song TJ Park JH Lee HS Nam CM Nam HS. Different prognostic value of white blood cell subtypes in patients with acute cerebral infarction. Atherosclerosis. (2012) 222:464–7. 10.1016/j.atherosclerosis.2012.02.042 22460048

[98] Buck BH Liebeskind DS Saver JL Bang OY Yun SW Starkman S. Early neutrophilia is associated with volume of ischemic tissue in acute stroke. Stroke. (2008) 39:355–60. 10.1161/STROKEAHA.107.490128 18162626

[99] Denorme F Manne BK Portier I Eustes AS Kosaka Y Kile BT. Platelet necrosis mediates ischemic stroke outcome in mice. Blood. (2020) 135:429–40. 10.1182/blood.2019002124 31800959 PMC7005363

[100] Agbani EO Poole AW. Procoagulant platelets: generation, function, and therapeutic targeting in thrombosis. Blood. (2017) 130:2171–9. 10.1182/blood-2017-05-787259 28972013

[101] Agbani EO van den Bosch MT Brown E Williams CM Mattheij NJ Cosemans JM. Coordinated membrane ballooning and procoagulant spreading in human platelets. Circulation. (2015) 132:1414–24. 10.1161/CIRCULATIONAHA.114.015036 26330411

[102] Jobe SM Wilson KM Leo L Raimondi A Molkentin JD Lentz SR. Critical role for the mitochondrial permeability transition pore and cyclophilin D in platelet activation and thrombosis. Blood. (2008) 111:1257–65. 10.1182/blood-2007-05-092684 17989312 PMC2214770

[103] Pang A Cui Y Chen Y Cheng N Delaney MK Gu M. Shear-induced integrin signaling in platelet phosphatidylserine exposure, microvesicle release, and coagulation. Blood. (2018) 132:533–43. 10.1182/blood-2017-05-785253 29853537 PMC6073322

[104] Senchenkova EY Ansari J Becker F Vital SA Al-Yafeai Z Sparkenbaugh EM. Platelet function to promote resolution of inflammation. Circulation. (2019) 140:319–35. 10.1161/CIRCULATIONAHA.118.039345 31154815 PMC6687438

[105] Chen Z Chopp M Zacharek A Li W Venkat P Wang F. Brain-Derived microparticles (BDMPs) Contribute to neuroinflammation and lactadherin reduces BDMP induced neuroinflammation and improves outcome after stroke. Front Immunol. (2019) 10:2747. 10.3389/fimmu.2019.02747 31993045 PMC6968774

[106] Bonaventura A Montecucco F Dallegri F. Cellular recruitment in myocardial ischaemia/reperfusion injury. Eur J Clin Invest. (2016) 46:590–601. 10.1111/eci.12633 27090739

[107] Yadav SS Howell DN Steeber DA Harland RC Tedder TF Clavien PA. P-Selectin mediates reperfusion injury through neutrophil and platelet sequestration in the warm ischemic mouse liver. Hepatology. (1999) 29:1494–502. 10.1002/hep.510290505 10216134

[108] Jansen MP Emal D Teske GJ Dessing MC Florquin S Roelofs JJ. Release of extracellular DNA influences renal ischemia reperfusion injury by platelet activation and formation of neutrophil extracellular traps. Kidney Int. (2017) 91:352–64. 10.1016/j.kint.2016.08.006 27692564

[109] Hirao H Nakamura K Kupiec-Weglinski JW. Liver ischaemia-reperfusion injury: a new understanding of the role of innate immunity. Nat Rev Gastroenterol Hepatol. (2021). 10.1038/s41575-021-00549-8. [Epub ahead of print]. 34837066

[110] Sayah DM Mallavia B Liu F Ortiz-Munoz G Caudrillier A DerHovanessian A. Neutrophil extracellular traps are pathogenic in primary graft dysfunction after lung transplantation. Am J Respir Crit Care Med. (2015) 191:455–63. 10.1164/rccm.201406-1086OC 25485813 PMC4351593

[111] Yuan Y Alwis I Wu MCL Kaplan Z Ashworth K Bark D Jr Pham A. Neutrophil macroaggregates promote widespread pulmonary thrombosis after gut ischemia. Sci Transl Med. (2017) 9:eaam5861. 10.1126/scitranslmed.aam5861 28954929

[112] Vaninov N. In the eye of the COVID-19 cytokine storm. Nat Rev Immunol. (2020) 20:277. 10.1038/s41577-020-0305-6 32249847 PMC7132547

[113] Malas MB Naazie IN Elsayed N Mathlouthi A Marmor R Clary B. Thromboembolism risk of COVID-19 is high and associated with a higher risk of mortality: a systematic review and meta-analysis. EClinicalMedicine. (2020) 29:100639. 10.1016/j.eclinm.2020.100639 33251499 PMC7679115

[114] Bonow RO Fonarow GC O'Gara PT Yancy CW. Association of coronavirus disease 2019 (COVID-19) with myocardial injury and mortality. JAMA Cardiol. (2020) 5:751–3. 10.1001/jamacardio.2020.1105 32219362

[115] Modin D Claggett B Sindet-Pedersen C Lassen Mats Christian H Skaarup Kristoffer G Jensen Jens Ulrik S. Acute COVID-19 and the incidence of ischemic stroke and acute myocardial infarction. Circulation. (2020) 142:2080–2. 10.1161/CIRCULATIONAHA.120.050809 33054349 PMC7682795

[116] Bonaventura A Vecchié A Dagna L Martinod K Dixon DL Van Tassell BW. Endothelial dysfunction and immunothrombosis as key pathogenic mechanisms in COVID-19. Nat Rev Immunol. (2021) 21:319–29. 10.1038/s41577-021-00536-9 33824483 PMC8023349

[117] Middleton EA He XY Denorme F Campbell RA Ng D Salvatore SP. Neutrophil extracellular traps contribute to immunothrombosis in COVID-19 acute respiratory distress syndrome. Blood. (2020) 136:1169–79. 10.1182/blood.2020007008 32597954 PMC7472714

[118] Manne BK Denorme F Middleton EA Portier I Rowley JW Stubben C. Platelet gene expression and function in patients with COVID-19. Blood. (2020) 136:1317–29. 10.1182/blood.2020007214 32573711 PMC7483430

[119] Ackermann M Verleden SE Kuehnel M Haverich A Welte T Laenger F. Pulmonary vascular endothelialitis, thrombosis, and angiogenesis in Covid-19. N Engl J Med. (2020) 383:120–8. 10.1056/NEJMoa2015432 32437596 PMC7412750

[120] Zaid Y Puhm F Allaeys I Naya A Oudghiri M Khalki L. Platelets can associate with SARS-CoV-2 RNA and are hyperactivated in COVID-19. Circ Res. (2020) 127:1404–18. 10.1161/CIRCRESAHA.120.317703 32938299 PMC7641188

[121] Althaus K Marini I Zlamal J Pelzl L Singh A Häberle H. Antibody-induced procoagulant platelets in severe COVID-19 infection. Blood. (2021) 137:1061–71. 10.1182/blood.2020008762 33512415 PMC7791311

[122] Pelzl L Singh A Funk J Witzemann A Marini I Zlamal J. Antibody-mediated procoagulant platelet formation in COVID-19 is AKT dependent. J Thromb Haemost. (2021) 18:1859–65. 10.1111/jth.15587 34752677 PMC8646637

[123] Geng X Mihaila R Yuan Y Strutt S Benz J Tang T. Inclacumab, a fully human anti-p-selectin antibody, directly binds to PSGL-1 binding region and demonstrates robust and durable inhibition of cell adhesion. Blood. (2020) 136 (Supplement 1):10–1. 10.1182/blood-2020-14053016462024

[124] Stähli BE Gebhard C Duchatelle V Cournoyer D Petroni T Tanguay JF. Effects of the P-selectin antagonist inclacumab on myocardial damage after percutaneous coronary intervention according to timing of infusion: insights from the SELECT-ACS trial. J Am Heart Assoc. (2016) 5:e004255. 10.1161/JAHA.116.004255 27852589 PMC5210344

[125] Tardif JC Tanguay JF Wright SR Duchatelle V Petroni T Grégoire JC. Effects of the P-selectin antagonist inclacumab on myocardial damage after percutaneous coronary intervention for non-ST-segment elevation myocardial infarction: results of the SELECT-ACS trial. J Am Coll Cardiol. (2013) 61:2048–55. 10.1016/j.jacc.2013.03.003 23500230

[126] Stähli Barbara E Tardif J-C Carrier M Gallo R Emery Robert W Robb S. Effects of P-Selectin antagonist inclacumab in patients undergoing coronary artery bypass graft surgery. J Am Coll Cardiol. (2016) 67:344–6. 10.1016/j.jacc.2015.10.071 26796402

[127] Ataga KI Kutlar A Kanter J Liles D Cancado R Friedrisch J. Crizanlizumab for the prevention of pain crises in sickle cell disease. N Engl J Med. (2017) 376:429–39. 10.1056/NEJMoa1611770 27959701 PMC5481200

[128] Sexton TR Zhang G Macaulay TE Callahan LA Charnigo R Vsevolozhskaya OA. Ticagrelor reduces thromboinflammatory markers in patients with pneumonia. JACC Basic Transl Sci. (2018) 3:435–49. 10.1016/j.jacbts.2018.05.005 30175268 PMC6115703

[129] Wallentin L Becker RC Budaj A Cannon CP Emanuelsson H Held C. Ticagrelor versus clopidogrel in patients with acute coronary syndromes. N Engl J Med. (2009) 361:1045–57. 10.1056/NEJMoa0904327 19717846

[130] Giles JA Greenhalgh AD Denes A Nieswandt B Coutts G McColl BW. Neutrophil infiltration to the brain is platelet-dependent, and is reversed by blockade of platelet GPIbalpha. Immunology. (2018) 154:322–8. 10.1111/imm.12892 29325217 PMC5979746

[131] Feagan BG Rutgeerts P Sands BE Hanauer S Colombel JF Sandborn WJ. Vedolizumab as induction and maintenance therapy for ulcerative colitis. N Engl J Med. (2013) 369:699–710. 10.1056/NEJMoa1215734 23964932

[132] Sandborn WJ Feagan BG Rutgeerts P Hanauer S Colombel JF Sands BE. Vedolizumab as induction and maintenance therapy for Crohn's disease. N Engl J Med. (2013) 369:711–21. 10.1056/NEJMoa1215739 23964933

[133] Wolf D Anto-Michel N Blankenbach H Wiedemann A Buscher K Hohmann JD. A ligand-specific blockade of the integrin Mac-1 selectively targets pathologic inflammation while maintaining protective host-defense. Nat Commun. (2018) 9:525. 10.1038/s41467-018-02896-8 29410422 PMC5802769

[134] Kelm M Lehoux S Azcutia V Cummings RD Nusrat A Parkos CA. Regulation of neutrophil function by selective targeting of glycan epitopes expressed on the integrin CD11b/CD18. FASEB J. (2020) 34:2326–43. 10.1096/fj.201902542R 31907993 PMC7018557

[135] Chen R Kang R Fan XG Tang D. Release and activity of histone in diseases. Cell Death Dis. (2014) 5:e1370. 10.1038/cddis.2014.337 25118930 PMC4454312

[136] Manfredi A Rovere-Querini P D'Angelo A Maugeri N. Low molecular weight heparins prevent the induction of autophagy of activated neutrophils and the formation of neutrophil extracellular traps. Pharmacol Res. (2017) 123:146–56. 10.1016/j.phrs.2016.08.008 28161237

[137] Holliday ZM Earhart AP Alnijoumi MM Krvavac A Allen LAH Schrum AG. Non-Randomized trial of dornase alfa for acute respiratory distress syndrome secondary to Covid-19. Front Immunol. (2021) 12:714833. 10.3389/fimmu.2021.714833 34745093 PMC8564175

[138] Gulick RM Lalezari J Goodrich J Clumeck N DeJesus E Horban A. Maraviroc for previously treated patients with R5 HIV-1 infection. N Engl J Med. (2008) 359:1429–41. 10.1056/NEJMoa0803152 18832244 PMC3078519

[139] Francisci D Pirro M Schiaroli E Mannarino MR Cipriani S Bianconi V. Maraviroc intensification modulates atherosclerotic progression in hiv-suppressed patients at high cardiovascular risk. A randomized, crossover pilot study. Open Forum Infect Dis. (2019) 6:ofz112. 10.1093/ofid/ofz112 30968058 PMC6446135

[140] Cipriani S Francisci D Mencarelli A Renga B Schiaroli E D'Amore C. Efficacy of the CCR5 antagonist maraviroc in reducing early, ritonavir-induced atherogenesis and advanced plaque progression in mice. Circulation. (2013) 127:2114–24. 10.1161/CIRCULATIONAHA.113.001278 23633271

[141] Joy MT Assayag EB Shabashov-Stone D Liraz-Zaltsman S Mazzitelli J Arenas M. CCR5 is a therapeutic target for recovery after stroke and traumatic brain injury. Cell. (2019) 176:1143–57. 10.1016/j.cell.2019.01.044 30794775 PMC7259116

